# Does methotrexate cause progressive fibrotic interstitial lung disease? A systematic review

**DOI:** 10.1007/s00296-020-04773-4

**Published:** 2021-01-29

**Authors:** Julie K. Dawson, Edmond Quah, Ben Earnshaw, Constanta Amoasii, Tareg Mudawi, Lisa G. Spencer

**Affiliations:** 1 Department of Rheumatology St Helens Hospital, St Helens and Knowsley Trust Hospitals, Marshalls Cross Road, St Helens, Merseyside UK; 2 Liverpool Interstitial Lung Disease Service, Aintree site, Liverpool University Hospitals NHS Foundation Trust, Liverpool, Lower Lane, Liverpool, L9 7AL UK

**Keywords:** Methotrexate, Progressive, Fibrotic interstitial lung disease, Methotrexate-induced pneumonitis

## Abstract

The aim is to evaluate the published evidence on whether methotrexate (MTX) use causes progressive fibrotic interstitial lung disease (fILD). This PRISMA-compliant systematic review has been registered electronically with PROSPERO 2018 ID CRD42018087838, Centre of review and dissemination at the University of York. A total of 29 articles met the inclusion criteria. Thirteen articles were found to support the claim that MTX causes fILD. They all had a low Downs and Black quality score (< 6/27). Their ‘risk of bias’ assessment scores indicated serious to critical risk of bias. The 16 articles rejecting the claim that MTX causes fILD were of higher quality as indicated by their Downs and Black score. Their ‘risk of bias’ assessment scores suggested only a low to moderate risk of bias. This systematic literature review supports the finding that MTX does not cause fILD in humans. Three studies suggest that MTX treatment may actually improve outcomes in patients with rheumatoid arthritis (RA) associated fILD by slowing down ILD progression.

**Supplementary Information:**

The online version contains supplementary material available at 10.1007/s00296-020-04773-4.

## Introduction

Over the years, multiple publications have appeared in the literature linking MTX use with various types of ‘lung injury’ including fILD. The first reported case of MTX exposure and lung ‘toxicity’ was published in 1969 in children treated with MTX for leukaemia [1]. Lung ‘complications’ have also been reported associated with its use in RA [2], psoriasis [3], and other cancers [4]. MTX thus gained a reputation of being ‘trouble’ for the lungs. On the specific issue, however, of whether MTX can cause a progressive fILD disease, there has been a long running debate particularly within rheumatology and respiratory medicine. Confusion as to whether MTX could cause fILD likely arose for two main reasons.

First, an historic inability to discriminate between the different forms of lung ‘toxicity’ that MTX can induce. MTX can indeed cause a type of ILD but this uncommon drug reaction is an inflammatory pneumonitis and not a truly fibrotic progressive ILD. Prior to detailed high-resolution computed tomography imaging (HRCT) and improved radiology reporting, identifying different types of ILD patterns was more difficult. With older, less advanced radiological imaging techniques one ILD pattern could easily appear to mimic another particularly on CXRs. Infection versus ILD can also be difficult to separate on occasions. Infective insults can also heal leaving a degree of static fibrotic damage. Patients may thus have been suspected to have developed fILD but in fact they did not have it.

Second, many RA patients receive MTX as all or part of their joint therapy. Fibrotic ILD has a disease intrinsic incidence of 4–7% in RA patients [5]. Suspecting that fILD may be causal to MTX use was thus not unreasonable. It is now perhaps better understood that fILD is a known extra articular complication of RA and is seen in RA patients who are completely treatment naïve from MTX.

It is perhaps best considered that MTX related ‘lung complications’ fall into two separate categories—infective and non-infective. Infective complications will not be covered in this review and are related to the drugs immunosuppressive actions. The non-infective complication is an acute event named MTX-induced pneumonitis.

**Fig. 1:**
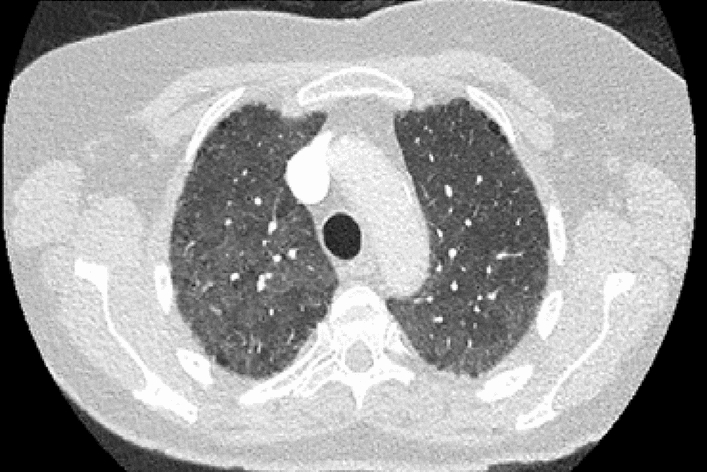
Transverse computed tomography (CT) image of chest showing diffuse ground glass inflammatory change in a case of MTXip in a patient with rheumatoid arthritis. Black areas are unaffected lung. There was no zonal predilection

MTX-induced pneumonitis (MTXip) is an acute inflammatory pulmonary complication. It is uncommon and reported to occur in 1% of RA patients commencing MTX [6]. It is associated with non-specific clinical features such as fever, shortness of breath, dry cough, hypoxia and negative cultures. Onset is usually within less than 12 months of MTX start-up [7]. Diagnosis of MTXip can be difficult due to its non-specific presentation. Several authors have proposed criteria to help confirm the diagnosis—Carson and Searles and Mckendry [8, 9]. HRCT changes include diffuse, bilateral ground glass change. (Fig. 1).

Lung histopathology often reveals cellular interstitial infiltrates, granulomas, or a diffuse alveolar damage pattern accompanied by perivascular inflammation. Fibrosis has been found in 29% of a histopathological (lung biopsy) series [10] although radiological fibrotic changes may not be evident on the HRCT. Lung biopsy is not mandated to make a firm diagnosis if the clinical picture fits. It should be noted that these HRCT and lung biopsy findings typically seen in MTXip are not exclusive to MTXip. They are also seen in other types of ILD, for example, in cases of hypersensitivity pneumonitis (HP). Hence, the HRCT changes seen in MTXip are often referred to as ‘HP type’ changes. Treatment is to promptly stop the MTX. Although not evidenced based, some may also give a short course of corticosteroids to ameliorate symptoms. If MTXip is not recognized with early cessation of MTX, it can be a life-threatening illness with a 20% mortality rate. It should be noted that the HRCT changes of a MTXip look little like the changes seen in RA fILD—an extra articular feature of RA in some patients.

**Fig. 2:**
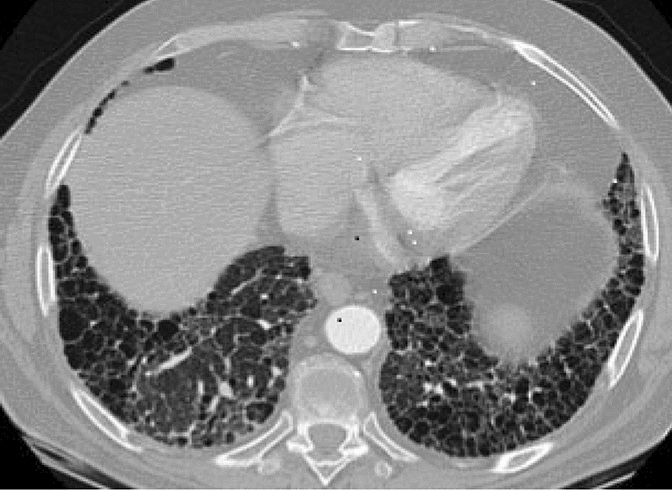
Transverse high-resolution computed tomography (CT) image of chest demonstrating a usual interstitial pneumonia (UIP) pattern—a fibrotic ILD pattern—in a patient with rheumatoid arthritis. Typically UIP pattern shows a basal predominance and emanates out from a subpleural location

Figure 2 shows typical HRCT findings in RA-related fILD which is far more common than MTXip. The fibrotic patterns seen in RA fILD usually fall into the ILD subgroups of usual interstitial pneumonia (UIP: most common pattern observed) and fibrotic non-specific pneumonia (fNSIP). Early fibrotic change can be missed in patients if HRCT imaging is not carried out at presentation. RA fILD develops insidiously, not acutely. However, acute exacerbations can occur with worsening symptoms of shortness of breath over weeks that can lead to more acute presentation [11]*.* Expert radiology reporting and clinical teams familiar with the various ILD patterns seen can fairly easily discriminate between RA-related fILD (with or without exacerbation) and a MTXip.

Over the last 20 years, our knowledge and understanding of the potential pulmonary complications of MTX and the natural history of RA has increased significantly. It is clear now having a diagnosis of RA leads to a disease specific, intrinsic risk of developing fILD. The previously accepted rationale that MTX could cause de novo fILD now needs reassessment. The significance of historical publications needs re-evaluating through a modern day lens. We thus report a critical analysis of the literature to identify if there is any high-quality evidence to conclude that MTX causes progressive fibrotic ILD when used in multiple disease areas as an immunosuppressant. We have uniquely used qualitative methods to analyse the studies to include the Downs and Black criteria [12] and risk of bias analysis. This we feel gives further depth to clinicians so they can tangibly understand the quality of the published papers. Whilst there have been meta-analysis of double blind randomized controlled MTX drug trials [13, 14] they are relatively short duration studies nor are they powered for rare events; thus, the need for observational real-world evidence. We also review the recent publications suggesting that MTX may slow the progression of RA associated f ILD.

## Methodology

An electronic literature search was completed in July 2020. The database used included PubMed, Cochrane central register, science direct and Google scholar. No date limit was set for studies searched. Keywords strings of ‘methotrexate’, ‘interstitial lung fibrosis’, ‘interstitial lung disease’, ‘fibrotic’, ‘methotrexate lung’, ‘methotrexate lung toxicity’, ‘long-term follow up methotrexate’ were used in this search. Full details of search terms are in supplement 1. We also examined cited papers in articles collected, to better understand the terms used in certain publications. The structure of this review was produced following the Preferred Reporting Items for Systematic Reviews and Meta-Analyses (PRISMA) checklist. The study has been registered electronically with PROSPERO 2018 ID CRD42018087838, Centre of review and dissemination at the University of York.

### Study selection

All study types were included—case reports, cohort studies, meta-analyses and systemic literature reviews without restriction. Two authors independently screened the titles and abstracts for eligibility. Where it was not clear from the abstract that the study was eligible, the paper was included in the full-text review. Any disagreements regarding abstract inclusion were resolved by a third independent reviewer.

Duplicated studies were removed. Inclusion and exclusion criteria are as below.

### Inclusion criteria

1. All studies with human subjects receiving MTX therapy
2. Articles that reviewed the complication of MTX-induced fILD

### Exclusion criteria

1. Studies using non-human subjects
2. All other MTX complications, including methotrexate-induced pneumonitis
3. Publications that examined the use of other pulmonary toxic agents apart from MTX
4. Abstracts

Abstracts of all collected articles were inspected then divided into two groups. One group contained studies that suggested a link between MTX and fILD. The other group contained studies that did not support a link. A meta-analysis of the data was not feasible because there were an insufficient number of patients with fILD associated with MTX therapy. Only qualitative analysis has been possible.

### Downs and Black analysis

Assessment questions are listed in supplement 2. A high score indicates high quality of the published intervention.

### Risk of bias analysis

Articles were assessed for risk of bias using the ‘The Risk Of Bias In Non-randomized Studies of Interventions’ (ROBINS-I) assessment tool [15].

## Results

A total number of 8638 hits were recorded before any studies were excluded. Pubmed: 3092 hits, Cochrane central register: 217 hits, Science direct: 4027 hits and Google scholar: 1302 hits. After de-duplication, we reviewed 1254 titles and abstracts; of this, we included 59 for full-text review. A further 30 were excluded after full review. The main reason for exclusion was that the study did not provide the relevant information to answer the study question. Flowchart 1 depicts the selection process for this review.
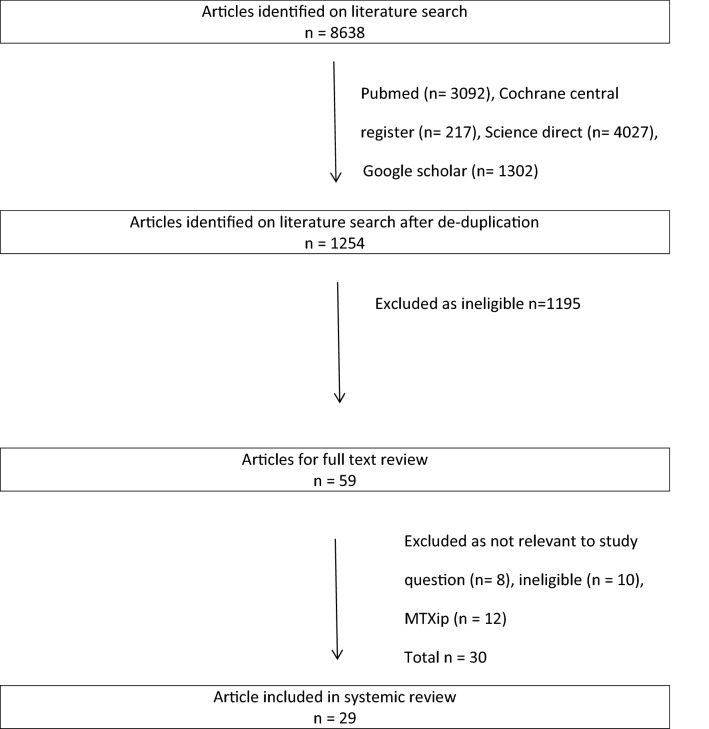


### Exclusion of studies on MTX-induced pneumonitis (MTXip)

Fifty nine studies were selected for further review. Of these, 18 articles were excluded, because they were found to be not relevant to the study question (*n* = 8) or ineligible (*n* = 10). A further 12 articles were removed because on more detailed assessment they actually described cases that fitted the criteria of MTXip not fibrosis. These articles used nonspecific terms describing the ‘lung complication’ such as ‘methotrexate-induced pulmonary complication’ or ‘methotrexate-induced lung toxicity’. In our review, we accessed referenced cited papers for each retrieved study to better understand the meaning of terms used. Articles that fulfilled the Carson [8] or Searles and McKendry [9] criteria for the diagnosis of MTXip were removed from this search for ‘reports on MTX causing fILD’ (not pneumonitis). This left 29 articles for final analysis.

### Type of studies

Six different study types were identified: systemic reviews of Randomised Controlled Trials (RCTs), narrative review article, cohort studies, case–control studies, case series and expert opinion. The majority of the studies were European, 13 (44%), followed by North America 10 (34%), Asia 3 (10%) and 1 study each from South America (4%), Africa (4%) and Australasia (4%).

### Accepting group (Table 1)

**Table 1:** Summary of the publications supporting that MTX causes fibrotic ILD

Authors	Publication year	Number of patients on MTX in study	Indication for MTX	Duration of study (years)	Possible cases of fILD	Oxford (UK) evidence grade	Downs and Black criteria score	ROBINS-I bias risk
Kaplan [15]	1978	1	Psoriasis	18	1	4	1/27	Critical
Willson [16]	1978	n/a	Neoplasm	n/a	n/a	5	0/27	n/a
Lewis [32]	1979	1	Psoriasis	17	1	4	2/27	n/a
Phillips [17]	1987	2	Psoriasis	6	1	4	4/27	n/a
Ali [18]	1998	92	RA	1	1	2b	4/27	Serious
Zitnik [33]	1990	n/a	n/a	n/a	n/a	5	0/27	Critical
Cannon [34]	1997	n/a	Rheumatic and non-rheumatic conditions	n/a	n/a	5	2/27	n/a
Di Muzio [35]	2000	n/a	n/a	n/a	n/a	5	0/27	n/a
Rossi [36]	2000	n/a	n/a	n/a	n/a	5	2/27	n/a
Schaller [37])	2010	n/a	RA	n/a	n/a	5	2/27	Critical
Atzeni [38]	2013	n/a	RA	n/a	n/a	5	1 /27	Critical
Hallowell [39]	2014	n/a	RA	n/a	n/a	5	0/27	Critical
Fredj [19]	2013	69	RA	n/a	2	2b	5/27	Critical

n/a = not applicable (information not found in paper)

It is noticeable there are very few original publications, only 13, in the accepting group once papers on methotrexate pneumonitis (MTXip) have been excluded. The supporting group consists of publications of case reports and review articles which are mainly low level of Oxford evidence grade. In terms of Downs and Black evidence quality the difference is even clearer with no high-quality scores from the publications that suggest methotrexate causes interstitial lung fibrosis.

As regards the three case reports they were published pre-1990 in patients taking MTX for psoriasis [16–18].

Ali et al. [19] found one patient developed fibrotic ILD in a RA cohort of 92 patients treated with MTX. These patients had failed to tolerate or respond to non MTX disease-modifying antirheumatic drugs (DMARDs) and had an average follow-up of 1 year. A comparator group of 32 patients with RA not treated with MTX did not have any cases of fibrotic lung disease. Authors attributed this ILD to either RA or MTX.

Fredj et al. [20] cross-sectionally studied pulmonary function tests (PFT) in patients whilst on MTX therapy for RA, 69/87 were treated with MTX, two patients had restrictive pattern PFT. They could not differentiate if this was related to RA or MTX toxicity. It was not clear if one or both patients were receiving MTX therapy.

The remaining eight articles are expert opinion-based review articles published from 1978 to 2014, they did not include any further additional original f ILD data.

### Rejecting group (Table 2)

**Table 2:** Summary of the publications rejecting that MTX causes fibrotic ILD

Authors	Publication year	Number of patients on MTX in study	Indication for MTX	Duration of study (years)	Cases of fILD in patients	Oxford (UK) evidence grade	Downs and Black criteria score	ROBINS-I bias risk
Wall [21]	1979	38	Osteogenic sarcoma	0.5	0	2b	13/17	Moderate
Furst [22]	1990	45	RA	3.3	0	4	19/27	Moderate
Barrera [23]	1994	350	RA/systemic sclerosis/ankylosing Spondylitis	8	0	2b	5/27	Low
Bedi [24]	1999	20	Psoriasis	1	0	2b	16/27	Moderate
Belzunegui [25]	2001	27	Psoriasis	4.3 Av 52 months	0	4	14/27	Moderate
Dawson [26])	2002	55	RA	2	11	2b	21/27	Low
Provenzano [27]	2003	18	RA	n/a	1	2b	11/27	Moderate
Conway [14]	2014	4544	RA	0.5–2	n/a	1a	17/27	Low
Conway [15]	2015	818	Psoriasis, psoriatic arthritis, inflammatory bowel disease	0.25–1	n/a	1a	16/27	Low
Iqbal [40]	2015	n/a	n/a	n/a	n/a	5	2/27	Moderate
Rojas-Serrano [28]	2017	52	RA-ILD	6	52	2b	21/27	Low
Kiely [29]	2019	1587	RA	25	39	2b	20/27	Low
Affleck [41]	2019	1	Psoriasis	10	1	4	3/27	n/a
Fragoulis [42]	2019	n/a	RA (field section of review)	n/a	n/a	5	14/27	Moderate
Juge [30]	2020	929	RA	n/a	126	2b	20/27	Moderate
Nokhatha [43]	2020	n/a	RA	n/a	n/a	5	3/27	Moderate

*n/a* not applicable (information not found in paper)

The 16 articles in the rejecting group contained larger studies with higher quality evidence and were at low and moderate risk of bias. The evidence base in the rejecting group was stronger overall. Articles included in the rejecting group were case reports, cohort studies, randomised controlled trials, HRCT based case–control study, two meta-analyses of randomised controlled MTX drug trials and review articles.

Wall et al. [21] investigated 38 children receiving up to 256 g/m^2^ of MTX for 3–8/12 therapy with vincristine in a paediatric oncology unit. PFT were undertaken before, during and after chemotherapy. One child developed diaphragmatic disease with lung volume and DLco drop. There were no changes in any child to suggest f ILD.

Furst et al. [22] reviewed long-term side effects of patients receiving MTX and included the details of 45 patients with RA that had moved onto open label studies in their unit. They specifically reported that they did not identify any f ILD.

Barera et al. [23] reviewed MTX pulmonary complications. This was predominantly a review on MTXip. They included data on 350 patients from MTX trials across several rheumatological conditions from their own unit. They reported MTXip, viral and atypical respiratory infections but no cases f ILD.

Bedi et al. [24] conducted pulmonary function tests on 20 patients who had taken 6 months of MTX for psoriasis. They did not find any significant deterioration of lung function in any of these patients.

Belzunegui et al. [25] undertook a cross-sectional study incorporating high-resolution computed tomography, with the results of PFT in 27 Caucasian patients with psoriatic arthritis treated with weekly low-dose MTX. In this cohort of patients, MTX was not associated with fibrotic ILD.

Dawson et al. [26] undertook a prospective study with initial HRCT and then serial PFT performed over a 2-year period on patients with Rheumatoid arthritis. Fifty patients received low-dose MTX and 73 patients were in a control group on no DMARD (nor alternative DMARD to MTX). On initial HRCT, 11 patients in MTX group had f ILD, 17 patients in the control group had fILD. This study showed no association between MTX and the development or progression of previously found fILD.

Provenzano [27] found no link between fILD and MTX use in RA patients. They investigated a cross section of patients for the presence of pulmonary disease with chest HRCT in 30 consecutive patients without respiratory symptoms and with normal chest X‐rays. Eighteen (60%) of patients had received low‐dose MTX. One patient had a pattern suggestive of fILD on HRCT pattern. They had never received MTX.

Conway et al. [14] undertook a meta-analysis of double blind, randomized controlled trials of MTX in patients with RA. A total of 22 studies with 8,584 participants were included and follow-up was for up to 2 years They demonstrated a small but significant increase in the risk of lung disease in patients with RA treated with MTX compared with other DMARD and biologic agents. The lung disease identified was nicely quantified but none of it was fILD. MTX was associated with an increased risk of all adverse respiratory events [Relative Risk (RR) 1.10, 95% confidence interval (95% CI) 1.02–1.19] and respiratory infection (RR 1.11, 95% CI 1.02–1.21). Patients treated with MTX were not at increased risk of death due to lung disease (RR 1.53, 95% CI 0.46–5.01) or noninfectious respiratory events (RR 1.02, 95% CI 0.65–1.60). A subgroup analysis of the studies in which pneumonitis was described, i.e. MTXip, revealed an increased risk associated with MTX use (RR 7.81, 95% CI 1.76–34.72).

Conway et al. [13] also investigated double blind randomized controlled trials of methotrexate versus placebo or active comparator agents in adults with psoriatic arthritis, psoriasis, or inflammatory bowel disease. Seven studies were included with 1630 participants. MTX was not associated with an increased risk of adverse respiratory events (RR 1.03, 95% CI 0.90–1.17), respiratory infections (RR 1.02, 95% CI 0.88–1.19), or non-infectious respiratory events (RR 1.07, 95% CI 0.58–1.96). No pulmonary deaths occurred.

Rojas-Serrano et al. [28] undertook a retrospective observational study of treatment of RA associated ILD (RA-ILD). Seventy eight patients were included. The types of ILD were-Usual Interstitial Pneumonia (UIP) 26%, Non-Specific Interstitial Pneumonia 36%, Lymphocytic Interstitial Pneumonia 19%, Cryptogenic Organising Pneumonia 5% and an overlap of ILD pattern types was seen in 36%. Fifty two patients with RA-ILD were treated with MTX specifically for ILD, 67% remained on MTX throughout study. There were more patients with UIP in the MTX treated group, only three patients with UIP were not treated with MTX. Treatment with MTX was associated with survival [hazard ratio (HR) 0.13, 95% CI 0.02–0.64]; *P* = 0.002. After adjusting for confounding variables, methotrexate was strongly associated with survival.

Two recent studies have looked at the incidence of ILD in patients with RA. Kiely et al. [29] investigated ILD specifically from case report forms in Early RA Study (ERAS) and the Early RA network (ERAN) cohort. Recruitment was from 1986 to 2012 with 2701 patients with up to 25-year follow-up. They had a control group of 1114 patients not exposed to MTX. Prevalence of ILD was 3.7%. They found no adverse association between RA-ILD and MTX use. Extended analysis, showed MTX exposure was associated with a reduced risk of developing incident ILD [Odds Ratio (OR) 0.48 *P* = 0.004] and longer time to ILD diagnosis (OR 0.41 *P* = 0.004). Juge et al. [30] undertook a case–control study in 410 patients with HRCT diagnosed with fibrotic RA-ILD and 673 patients with RA without ILD on HRCT. The combined estimate revealed an adjusted OR of 0.43 (95% CI 0.26–0.69; *P* = 0.0006) in MTX ever users for ILD. ILD detection was significantly delayed in MTX ever users compared to never users (11.4 ± 10.4 years and 4.0 ± 7.4 years, respectively; *P* < 0.001). MTX use was not associated with an increased risk of RA-ILD in patients with RA, and in fact ILD was detected later in MTX treated patients.

From 2015 onwards, review articles written by Rheumatologists moved over to the rejecting group and started raising the point that there was no good evidence that MTX causes nor exacerbates underlying RA-ILD.

Following the completion of our literature search, in August 2020, a randomised double blinded placebo controlled trial was published investigating if low-dose MTX prevented cardiovascular disease events [31]. It reported pulmonary adverse events. The study was suspended due to lack of efficacy after 4786 participants had been recruited. Median follow-up was 23 months, 2391 participants were assigned to MTX, all participants had normal baseline CXR within 12 months of enrolment into the study. Pulmonary adverse events were increased in the MTX group [HR 1.52 (95% CI 1.16–1.98)] The events were reported as possible pneumonitis, shortness of breath and bronchitis.

There were seven possible pneumonitis cases in MTX group and one in the placebo group. This accounted for 0.17% of the participants. The difference between groups did not reach statistical significance. Searles and McKendry criteria were used for diagnosing MTXip. Any pulmonary adverse event with a mention of pulmonary fibrosis, interstitial lung disease, or ground glass opacities was reviewed for the possibility of pneumonitis.

Of the seven patients in the MTXip group, five out of the seven had onset of symptoms over 12 months from commencing MTX. One patient in the MTX group and one in the control group were on amiodarone, a cardiac medication also known to cause ILD. Two patients had nodular infiltrates on CT scanning which is described in MTXip. In one of the patients the respiratory condition could have be sarcoidosis or MTXip. Two patients in the MTX group were described as having features that we would classify as fibrotic ILD from CT or biopsy findings. This was not statistically significant and is in keeping with the incidence of fibrotic ILD (0.9%) [5]. This paper does not provide any evidence that MTX causes lung fibrosis.

## Discussion

We have demonstrated that the published evidence base supporting the concept that MTX is causally linked to the development of progressive fibrotic ILD is weak. The evidence against MTX being linked to the development of fibrotic ILD is much stronger.

Historically the pattern has been that as more clinical cases of MTXip were described, a theme to publish papers where fibrosis was found in addition to pneumonitis changes evolved. Sostman et al. [4] had described MTXip cases with diffuse pneumonitis infiltrate and mild fibrosis in their series in two of their oncology cases. Bedroissan et al. [3] found fibrotic changes post-mortem in three patients where MTX was used for oncology and psoriasis indications. Van de Veen [44] described this in RA. Subsequent histopathological review papers have confirmed fibrosis is found on biopsy in 29% of patients with MTXip, typically without honeycombing [10]. This influenced the summary of product information for methotrexate as well as terminology in the titles of the papers and review articles published. This of course has then had a knock on effect in national formularies and educational literature.

Of note within the supporting review articles Cannon [34] proposed there were five types of lung disease caused by MTX one of which was interstitial fibrosis. He observed that the case reports of pulmonary fibrosis related to MTX were in patients with psoriasis, a condition thought not to be associated with underlying interstitial lung disease. He thus thought that it was reasonable to conclude that methotrexate caused interstitial fibrosis. Applying todays knowledge of idiopathic interstitial lung disease and drug induced lung disease a different conclusion may well have been drawn. On closer scrutiny the only case of probable fibrotic ILD it references is the Kaplan publication [16]. The rest are MTXip cases where either the term fibrosis rather than pneumonitis was used or histologically fibrosis was found in addition to methotrexate pneumonitis changes i.e. a finding still in keeping with MTXip.

Decades later an interchange of letters, related to Affleck et al. [41] case report, were published. This case is very similar to Kaplan’s but the authors strongly believed the patient died of Idiopathic Pulmonary Fibrosis not MTX-induced fibrosis. When we consider cases of drug induced interstitial lung disease (DIID) we should now consider the American Thoracic/European Respiratory (ATS/ERS) consensus classification criteria [45]. DIID is characteristically defined as the lung disease being closely temporally related to the medication and demonstrating response to withdrawal of the drug, with exclusion of infection and an alternative cause for the ILD. This is in keeping with criteria Carson proposed but is more exacting than Searles and Mckendrys criteria. Objective improvement on withdrawal of MTX is actually lacking in the supporting published psoriasis case reports. Searles and McKendry criteria which have been widely used, however, have never been validated and have set a low threshold for MTXip.

We also suggest that earlier publications, in error, linked the natural presence of RA-related fILD to the use of MTX. We suspect that historically there was an inability to accurately discriminate between different types of lung injury that MTX can induce. This may have led to the clinical picture becoming muddled in some cases. Two recent studies have shown MTX reduces the risk of developing fILD, delaying its onset [29, 30] and one found MTX treatment was clinically and statistically associated with improved survival [28]. These findings are very much against MTX causing or exacerbating ILD in patients with RA and in fact may indicate some sort of protective mechanism afforded by use of MTX at least in RA f ILD cohorts.

It is notable that MTX is also used in many other diseases to dampen down inflammation. Its use in patients with granulomatosis polyangiitis [46] and sarcoidosis [47] has not been associated with any reports of de novo fILD development. In pulmonary sarcoidosis MTX monotherapy has in fact been shown to improve lung function [48].

### Strengths and limitations of review

As far as we are aware, this is the first review focussing only on whether MTX causes progressive fibrotic ILD. The inclusion of all studies that involved the use of MTX regardless of the disease being treated has provided a broader and perhaps clearer picture.

Errors may have been introduced in our analysis due to the way we interpreted the variable terminology used in articles to describe their pulmonary complications. For example, we may have excluded an article because we felt, by today’s standards; it described a case of MTXip rather than true fILD. We may thus have excluded important information. We attempted to minimize this by applying the available MTXip criteria to our analysis. Where the relevant information was provided in the articles we used the more robust Carson criteria which requires information about histopathological confirmation of the MTXip diagnosis and information about any response to MTX withdrawal. In all other cases, the less specific Searles and McKendry criteria were applied. In addition some patients in the articles assessed were often on a number of disease-modifying drugs (DMARDS) not just MTX—particularly studies reporting on RA cohorts. DMARDS other than MTX can cause lung complications. Any literature review is dependent on the detail given in the publications for our ability to differentiate the true cause of any pulmonary effects reported.

## Conclusion

We have demonstrated that the published evidence base supporting the concept that MTX is causally linked to the development of progressive fibrotic ILD is weak. The evidence against MTX being linked to the development of progressive fibrotic ILD is much stronger. In addition, although not part of the hypothesis of this study, we found high-quality evidence that patients with RA-related fILD, who were treated with MTX, actually had increased survival. This suggests a beneficial rather than harmful effect of MTX on the lungs. Prospective research studies would be required to further assess this retrospective observation.

To avoid future possible misclassification of any lung complications due to MTX, we advise close working with respiratory physicians with expertise in ILD and the use of a multidisciplinary team meeting to discuss cases.

We hope our review will be widely disseminated and will lead to fewer patients having their MTX stopped due to a finding of fibrotic ILD intrinsic to their underlying RA. Clinicians still need to remain vigilant, however, to the development of other pulmonary side effects from MTX including rare cases of MTX-induced pneumonitis and infections.

## Supplementary Information

Below is the link to the electronic supplementary material.

Supplementary file1 (DOCX 13 KB)

Supplementary file2 (DOCX 16 KB)
