# Supplementary material for: Does methotrexate cause progressive fibrotic interstitial lung disease? A systematic review
Source: Rheumatol Int. 2021 Jan 29;41(6):1055–64. doi: 10.1007/s00296-020-04773-4 (PMC8079289; doi:10.1007/s00296-020-04773-4)
Supplement: Supplementary file 1 — Supplementary file1 (DOCX 13 KB) [file 296_2020_4773_MOESM1_ESM.docx]

Supplement 1

MESH Terms:

[ Lung disease, Interstitial (exp), Pulmonary Fibrosis (exp), Pulmonary Injury (exp), lung fibrosis (exp), lung injury (exp)]

AND

[ Antimetabolites, Antineoplastic (exp), Folic acid antagonists (exp), methotrexate]

Keyword searches:

(Methotrexate) OR (folate antagonist) OR (antimetabolite)

AND

(lung fibrosis) OR (Interstitial lung fibrosis) OR (pulmonary fibrosis) OR ( Interstitial lung disease) OR ( Interstitial lung injury) OR ( Lung Injury) OR ( methotrexate Lung disease) OR ( Methotrexate lung injury) OR ( methotrexate lung toxicity) OR (fibrotic lung) OR (progressive lung fibrosis)
